# Nectin-4 is a new histological and serological tumor associated marker for breast cancer

**DOI:** 10.1186/1471-2407-7-73

**Published:** 2007-05-02

**Authors:** Stéphanie Fabre-Lafay, Florence Monville, Sarah Garrido-Urbani, Carole Berruyer-Pouyet, Christophe Ginestier, Nicolas Reymond, Pascal Finetti, Richard Sauvan, José Adélaïde, Jeannine Geneix, Eric Lecocq, Cornel Popovici, Patrice Dubreuil, Patrice Viens, Anthony Gonçalves, Emmanuelle Charafe-Jauffret, Jocelyne Jacquemier, Daniel Birnbaum, Marc Lopez

**Affiliations:** 1 Inserm, UMR599, Centre de Recherche en Cancérologie de Marseille, Marseille, F-13009, France; 2 Univ. Méditerranée, Marseille, F-13007, France; 3 Institut Paoli-Calmettes, Marseille, F-13009, France; 4 Institut Paoli-Calmettes, Laboratoire d'oncologie moléculaire, Marseille, F-13009, France; 5 Institut Paoli-Calmettes, Département d'oncologie médicale, Marseille, F-13009, France; 6 Centre d'Immunologie Pierre Fabre, St Julien en Genevois, F-74164, France; 7 Comprehensive Cancer Center, University of Michigan, Ann Arbor, Michigan 48109, USA; 8 Ludwig Institute for Cancer Research, London W1W 7BS, UK

## Abstract

**Introduction:**

Breast cancer is a complex and heterogeneous disease at the molecular level. Evolution is difficult to predict according to classical histoclinical prognostic factors. Different studies highlight the importance of large-scale molecular expression analyses to improve taxonomy of breast cancer and prognostic classification. Identification of new molecular markers that refine this taxonomy and improve patient management is a priority in the field of breast cancer research.

Nectins are cell adhesion molecules involved in the regulation of epithelial physiology. We present here Nectin-4/PVRL4 as a new histological and serological tumor associated marker for breast carcinoma.

**Methods:**

Expression of Nectin-4 protein was measured on a panel of 78 primary cells and cell lines from different origins and 57 breast tumors by FACS analysis and immunohistochemistry (IHC), respectively. mRNA expression was measured by quantitative PCR.

Serum Nectin-4 was detected by ELISA and compared with CEA and CA15.3 markers, on panels of 45 sera from healthy donors, 53 sera from patients with non-metastatic breast carcinoma (MBC) at diagnosis, and 182 sera from patients with MBC. Distribution of histological/serological molecular markers and histoclinical parameters were compared using the standard Chi-2 test.

**Results:**

Nectin-4 was not detected in normal breast epithelium. By contrast, Nectin-4 was expressed in 61% of ductal breast carcinoma vs 6% in lobular type. Expression of Nectin-4 strongly correlated with the basal-like markers EGFR, P53, and P-cadherin, and negatively correlated with the luminal-like markers ER, PR and GATA3. All but one ER/PR-negative tumors expressed Nectin-4. The detection of Nectin-4 in serum improves the follow-up of patients with MBC: the association CEA/CA15.3/Nectin-4 allowed to monitor 74% of these patients compared to 67% with the association CEA/CA15.3. Serum Nectin-4 is a marker of disease progression, and levels correlate with the number of metastases (*P *= 0.038). Serum Nectin-4 is also a marker of therapeutic efficiency and correlates, in 90% of cases, with clinical evolution.

**Conclusion:**

Nectin-4 is a new tumor-associated antigen for breast carcinoma. Nectin-4 is a new bio-marker whose use could help refine breast cancer taxonomy and improve patients' follow-up. Nectin-4 emerges as a potential target for breast cancer immunotherapy.

## Introduction

Breast cancer is the most common malignancy in women and will affect around one in 10 women during their lifetime. Breast cancer is a heterogeneous disease at the molecular level and treatment is efficient in only 70% of cases [1]. Identification of new molecular tumor associated biomarkers is one the most important current challenge [2]. Tumor associated molecular markers can be useful to improve the detection, taxonomy, diagnosis, prognosis, follow-up, and therapy of breast cancer.

Individual molecular markers and patterns of markers subdivide traditional breast cancer classes into subsets that behave as useful taxonomic and prognosis entities. ERBB2 is the prototypic individual marker: it is a marker of adverse prognosis, and the serum detection of a soluble form is used in the follow-up of breast cancer patients. It is also a therapeutic target. Beside ERBB2 overexpressing breast tumors, four other subtypes (luminal A, luminal B, basal and normal-like) have been described according to patterns of marker expression using DNA and tissue microarrays [3-6]. Luminal-A and basal breast cancers correspond to two very distinct epithelial subtypes. They show different clinical course and response to therapy [4-8]. However, this classification lacks precision as judged by the adverse evolution of many patients with apparent good prognosis and vice versa. Thus, there is a need to define new markers to refine histoclinical and molecular classifications and improve breast cancer management.

We have recently characterized a new family of cell adhesion molecules homologous to PVR/CD155 (the PolioVirus Receptor) named Nectins [9-12]. Nectins are members of the immunoglobulin superfamily (IgSF) and are components of E-cadherin-based adherens junctions in epithelial cells. Four Nectins have been described so far. They are structurally related and exhibit three conserved immunoglobulin-like domains (V, C, C) in their extracellular regions. Nectins and E-cadherin are linked to F-actin through AF-6/Afadin and Catenins, respectively [13]. The Nectin/Afadin and E-cadherin/Catenins systems interact with each other through Afadin and α-catenin. Nectins and Afadin have been involved in tumor biology. i) PVR and Nectin-2 have been described as tumor antigens and are molecular targets of NK cells through the interaction with the DNAM-1/CD226 molecule [14], ii) PVR is overexpressed in tumors of hematopoietic, neuronal and epithelial origins [15-17], iii) Nectin-1 is overexpressed is squamous carcinomas [18], and iv) Afadin is ubiquitously expressed in epithelia, and its loss has been associated with poor outcome in breast carcinoma [19].

All Nectins except Nectin-4 are expressed in epithelial, endothelial, hematopoietic and neuronal cells in adult tissues [12]. Nectin-4 is mainly expressed during embryogenesis but is not detected in normal adult tissues nor in serum.

In the present study, we show that Nectin-4 is a new tumor-associated antigen and a reliable marker for breast carcinoma. Nectin-4 is indeed not detected in normal breast epithelial cells, and highly expressed both in tumor cell lines and tumors from breast origin. Nectin-4 expression profile correlates with the expression of markers that define the basal subtype in breast tumors. Finally, Nectin-4 is shed from the tumor cell surface and represents a sensitive, reliable, and complementary serum marker for the follow-up of patients with metastatic breast carcinoma.

## Materials and methods

### Patients and histological samples

Liquid nitrogen flash-frozen breast cancer samples were randomly selected from consecutive series of more than 650 ductal and 110 lobular breast carcinoma samples. A panel of 57 tumor samples at the time of diagnosis and prior any adjuvant therapy was obtained from women treated at the Institut Paoli-Calmettes, Marseille, France. Consecutive series of 26 ductal and 31 lobular carcinoma were selected to correlate Nectin-4 expression with the histological type. Tumors were classified according to the WHO classification. The histoprognostic grade used was the modified Scarf Bloom Richardson (SBR) grading for invasive lesions. Five normal breast samples were obtained from healthy women who had undergone reductive mammary surgery.

### Patient serum selection

Several panels were selected: Series of 45 sera from healthy female donors, without evidence of breast cancer; 53 sera from patients with non MBC at diagnosis who were not receiving any therapy; 182 sera from patients with MBC (81% ductal, 19% lobular): 70 sera corresponding to synchronous MBC patients and 112 sera to metachronous MBC patients, 64 of which with samples collected before and after specific anticancer treatment. For each panel, selection was random. Analyses of other specific biomarkers, CEA and CA15.3 were done on these sera.

This study was approved and executed in compliance with our Institutional Review Board.

### Cells and culture conditions

Human leukocytes were purified from healthy donors using Ficoll separation. Human umbilical vein endothelial cells (HUVEC) were isolated and cultivated as previously described [20]. CD34 positive cells were purified with magneted activated cell sorter (MACS) as already reported [21]. Hematopoietic cell lines were cultivated in RPMI medium supplemented with 10% foetal calf serum. The 31 breast tumor cell lines have been previously described [22]. The bladder tumor cell lines were kindly provided by Dr. F. Radvanyi (Institut Curie, Paris, France). Ovarian O114, O151 and O170 cell lines were kindly provided by Pr V. Catros (CHU de Rennes, France). Ovarian TOV-21G, TOV-112D and OV-90 were a gift from Pr. Mes-Masson (Institut du Cancer de Montréal, Canada) [23]. IGR-OV1 was from Institut Gustave-Roussy, Paris, France. The other cell lines were from ATCC (Manassas, VA, USA). All cell lines were grown according to the recommendations of the supplier, in an air – 5% CO_2 _atmosphere at constant humidity.

### RNA extraction

Total RNA was extracted from frozen cell lines as previously described [24]. RNA integrity was controlled by electrophoresis on agarose gels and by Agilent micro-analysis (Agilent Bioanalyzer, Palo Alto, USA).

### Quantitative PCR

Complementary DNA (cDNA) was synthesized from 2 μg total RNA using SuperScript™ III Reverse Transcriptase kit (Invitrogen, France). Expression of *Nectin4 *and *GUSB *(located at 7q21.11 and used for normalisation of expression data) was measured using the LightCycler^®^FastStart DNA Master^PLUS ^SYBR Green I kit in the Light Cycler^® ^2.0 instrument (Roche, Germany), as recommended by the supplier.

The expression of *Nectin4 *and *GUSB *(located at 7q21.11 and used for normalisation of expression data) was measured using the LightCycler^®^FastStart DNA Master^PLUS ^SYBR Green I kit in the Light Cycler^® ^2.0 instrument (Roche, Germany), as recommended by the supplier.

Relative quantitative analysis of the expression of *Nectin 4 *gene was performed with serially diluted samples of EcoRI linearised plasmid containing the ORF cloned in the pFLAG-CMV25 vector (Sigma). Nectin-4 primers were defined using the "primer3" algorithm. Two primers were selected: huN4S: CAAAATCTGTGGCACATTGG and huN4AS: GCTGACATGGCAGACGTAGA leading to an amplified product of 189 bp. For the relative quantitative analysis of *GUSB *gene, serially diluted samples were purchased from Ipsogen (Marseille, France).

The relative expression level of *Nectin4 *in each sample was calculated by normalizing the corresponding expression with G*USB *expression values.

### Antibodies for Nectin-4

Anti-Nectin-4 monoclonal antibodies (mAbs) were obtained after successive intraperitoneal injections of BALB/c mice with 20 μg of human recombinant soluble Nectin-4 (N4VCC)-Fc [25]. Two hundred clones were tested for differential reactivity to COS cells and COS cells transiently expressing human Nectin-4. MAbs were then tested for cell surface detection of Nectin-4 by FACS analysis and for their ability to react with soluble recombinant Nectin-4, N4VCC-Fc and N4V-Fc (Nectin-4 deleted from its 2 IgC domains) by ELISA (see below). Two clones were thus selected: N4.61 clone is specific of the IgV domain and N4.40 clone is specific of one of the two IgC domains [25]. These mAbs did not recognize Nectin-1, Nectin-2, Nectin-3 nor PVR (data not shown).

### Cell surface expression analysis of Nectin-4

2 × 10^5 ^cells were incubated for 60 min at +4°C with 10 μg/ml of either N4.40 or N4.61 mAbs, washed, and then revealed by incubation for 45 min at +4°C with a phycoerythrin labelled goat anti-mouse antibody (Immunotech, France). Samples were processed by FACS analysis. Mean Fluorescence Intensity (MFI) value of each sample were measured and normalized by the MFI value of isotypic control.

### Selection of markers

Proteins were selected according to i) their previously described role as breast cancer taxonomic immunohistochemical markers, i.e., hormone receptors (ER and PR), ERBB2, EGFR, P53, BCL2, GATA3, P-cadherin [22,26,27], and ii) their functional relevance to Nectin biology, i.e., E-cadherin, β-catenin and AF-6/afadin [11-13]. Characteristics of the antibodies and experimental conditions were previously described [22].

### Immunohistochemical analysis (IHC)

For Nectin-4, IHC was carried on 5 μm sections from frozen tissue. Sections were fixed in acetone for 10 min, air-dried for 10 min and rehydrated in TBST (Dako, Coppenhagen, Denmark). Staining was done at room temperature and DAKO EnVision™ System was used to reveal the staining. Slides were first incubated with the primary antibody N4.61 or N4.40 (0.5 μg/ml) for 30 min. After washes in TBST, slides were incubated with secondary antibodies (anti-mouse) conjugated with alkaline phosphatase for 30 min. Fast Red substrate-chromogen solution was prepared and used as dye. Slides were counter-stained with haematoxylin, and mounted on slide using Aquatex (Merck, Darmstadt, Germany).

For the other markers, IHC was carried on tissue microarrays prepared as previously described. They were deparaffinized in Histolemon (Carlo Erba Reagenti, Rodano, Italia) and rehydrated in graded alcohol. Antigen enhancement was done by incubating the sections in citrate buffer pH 6 (Dako, Coppenhagen, Denmark) as recommended. Slides were then transferred to a Dako autostainer. Staining was done at room temperature as follows. After washes in phosphate buffer, followed by quenching of endogenous peroxidase activity by treatment with 0.1% H_2_O_2_, slides were first incubated with blocking serum (Dako) for 10 min and then with the primary antibody for one hour. After washes, slides were incubated with biotinylated secondary antibody for 20 min followed by streptavidin-conjugated peroxidase (Dako LSAB^R^2 kit). Diaminobenzidine was used as the chromogene. Slides were counterstained with haematoxylin, and mounted on slides using Aquatex (Merck, Darmstadt, Germany) mounting solution. Slides were evaluated under a light microscope. Immunoreactivities were classified by estimating the quick score (Q) as previously described [22]. ERBB2 status was calculated with the Dako scale (Herceptest™ kit scoring guidelines). Quick score allowed the separation of tumors into two or three classes. Homogeneous classes were defined by grouping samples with an equivalent staining level according to the distribution curves as described. Negative and positive classes were defined for Afadin, α and β catenins. EGFR, GATA3, P53, P-cadherin, and PR were defined with a positivity cut-off value of Q = 1. Positivity cut-off value for MIB1/Ki67 and Nectin-4 was Q = 20. Three classes were defined (negative (Q = 0), moderate (0 < Q ≤ 100) and strong staining (100 < Q ≤ 300) for E-cadherin and ER. For ERBB2 three classes (0/1+, 2+, 3+) were defined according to the Dako scale. Corresponding antibodies have been previously described [26]. Unsupervised hierarchical clustering was used to investigate relations between samples and between proteins as previously described [22].

### Statistical methods

Distribution of histological/serological molecular markers and histoclinical parameters were compared using the standard Chi-2 test.

### ELISA

Nectin-4 ELISA has been previously described [28]. Briefly, a sandwich enzyme-linked immunosorbent assay (ELISA) was used to detect soluble Nectin-4 in patients' sera. Ninety-six-well trays were coated with N4.40 at 10 μg/ml. After saturation of wells with phosphate-buffer-saline containing 1% bovine serum albumin (PBS-1%BSA), 100 μl of culture medium or serum was incubated for 12 h at 4°C, then with 2.5 μg/ml biotinylated mAb N4.61 for 2 h at 37°C. Streptavidin-peroxidase (2 μg/ml) in PBS-1%BSA was incubated for 1 h at 37°C. One hundred μl of peroxidase substrate was added (One Step ABTS, Pierce) and optical density was read at 405 nm. Three washing steps were performed between each incubation with 100 μl PBS containing 0.5% Tween 20, except for the last step (5 wash steps). Duplicates were analyzed and mean values were calculated. For each determination, threshold value was defined by the mean level value of 10 normal sera and was generally below 30 pM. Serum above the threshold values (>30 pM), were considered as positive. Nectin-4 concentration in sera was calculated using serial dilutions of soluble recombinant N4VCC-Fc (concentrations ranging from 1 × 10^-9 ^M to 7.5 × 10^-12 ^M). This ELISA does not detect soluble Nectin-1, -2, -3, and PVR-Fc used at the same concentrations. Sensitivity of the test is 7.5 pM. Measurement is linear up to 10^-9^M.

### Receiving Operator Characteristic (ROC) analysis

To determine the accuracy of a diagnosis approach using CEA, CA15.3 and Nectin-4 markers, receiver operating characteristic (ROC) curves were produced for the four following associations: CEA+CA15.3+Nectin-4, CA15.3+N4, CEA+N4, CEA+CA15.3. A series of 182 sera of MBC patients were considered for this study. Sensitivity, specificity and areas under ROC curves were calculated in each case.

## Results

### Nectin-4 is prominently expressed in breast tumor cell lines

We previously cloned human and murine Nectin-4. Northern blot analyses showed that Nectin-4 is expressed in mouse embryo from day 11 d.p.c. Expression of Nectin-4 differs between mouse and human adult tissues. Whereas Nectin-4 expression was detected in brain, lung, and testis in the mouse, it was only found in placenta and slightly in trachea among 23 human tissues tested [12].

**Figure 1:**
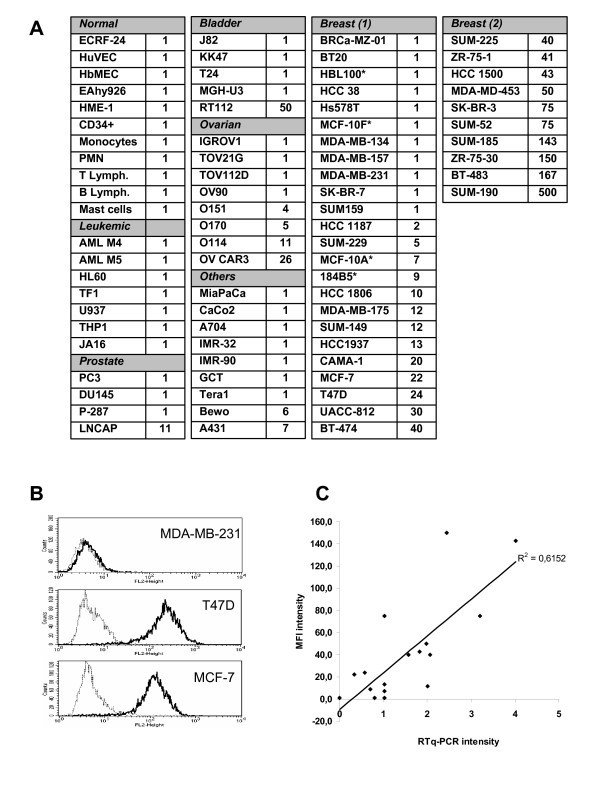
**Analysis of Nectin-4 expression in normal and tumor cells**. **A: **Cell surface expression of Nectin-4 was assessed by FACS analysis using the N4.61 mAb. The level of cell surface expressed Nectin-4 (L) was calculated in arbitrary units based on fluorescence intensity: L = MFI N4.61/MFI control IgG1. Similar results were obtained with the N4.40 mAb. L = 1 corresponds to negative cell lines, 2 < L < 15: low expression, 16 < L < 50: intermediate expression, L > 50 high expression. These results are representative of at least three independant experiments. *, non cancerous breast cell lines. **B: **Nectin-4 expression level on breast tumor cell lines was monitored by FACS analysis using the anti-Nectin-4 N4.61 mAb (black line) and compared with a mouse irrelevant IgG1 (gray line). Examples of a negative cell line (MDA-MB-231) and two intermediate expressing cell lines (T47D and MCF-7). **C: **Correlation between Nectin-4 cell surface expression and Nectin-4 transcriptional expression. Quantitative PCR was performed on 17 breast tumor cell lines. Values were calculated as described in material and methods. The results are representative of at least two experiments.

To extend this study, we analyzed the expression of Nectin-4 in a panel of primary cells and tumor cells from different origins (Fig. 1A) using two monoclonal antibodies (mAbs) (N4.40 and N4.61) [25,28] and FACS analysis. Nectin-4 was not expressed in normal cells, including endothelial (ECRF-24, HUVEC, HbMEC, Eahy926), epithelial (HME-1), and hematopoietic cells (bone marrow progenitors, monocytes, PMN, T and B lymphocytes, mast cells). Expression of Nectin-4 in tumor cell lines varied according to the origin of the tumor: No expression was detected in leukemic cells. Nectin-4 was expressed in one of four cell lines derived from prostate (LnCaP) and bladder (RT112) carcinomas. Half of ovarian tumor cell lines expressed Nectin-4. Nectin-4 was expressed in the epidermoid carcinoma cell line A431 and the choriocarcinoma cell line BeWo. Nectin-4 expression was detected at various levels in 68% of breast cell lines (23/34). No or moderate expression was detected in non-cancerous cell lines (HME-1, MCF-10F, MCF-10A and 184B5). Nectin-4 expression pattern is usually homogenous as exemplified in Fig. 1B. Even though the characterization and specificity of N4.40 and N4.61 mAbs have already been reported, quantification of Nectin-4 specific transcripts was undertaken by quantitative PCR and compared to cell surface expression. The results show a correlation (R^2 ^= 0.6145) between transcript and protein levels and strenghten the accuracy and reliability of the mAbs for the quantification of Nectin-4 expression. These data demonstrate that Nectin-4 is not expressed in normal cells and that Nectin-4 is prominently expressed in ovarian and above all, in breast tumor cell lines.

### Nectin-4 is prominently expressed in ductal breast carcinoma

We then studied Nectin-4 expression in normal breast epithelium and breast carcinoma. Ductal and lobular carcinoma account for around 80% and 15% of breast cancers, respectively. For each of these histologic types, specimens were randomly selected from our resources and analyzed by IHC for Nectin-4 expression.

**Figure 2:**
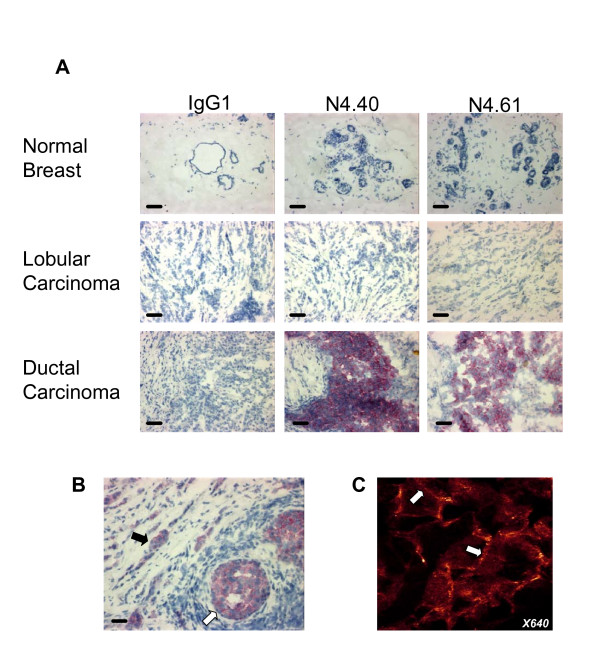
**Nectin-4 expression in breast carcinoma**. **A: **Normal breast epithelium does not express Nectin-4. No expression was detected in luminal, myoepithelial and stromal cells. Nectin-4 expression was found prominently in ductal carcinoma. Bar 50 μm. Magnification: × 200. **B: **Both invasive (black arrow) and in situ (white arrow) components of ductal carcinoma are immunostained by Nectin-4. **C: **Immunofluorescence analysis revealed that Nectin-4 expression is cytoplasmic (arrowhead) and junctional (arrow). Similar results were obtained with both mAbs Magnification: × 640.

Nectin-4 was not detected in sections of normal breast tissues, including luminal, myoepithelial, stromal and endothelial cells (Fig. 2A). Expression was detected in 62% of ductal type and 6% of lobular type carcinoma strengthening our results obtained with cell lines. Nectin-4 expression thus strongly correlates with histological type (see Table 1). As examplified in Fig. 2B, Nectin-4 was expressed both in non-invasive (white arrow) and invasive (black arrow) tumors. Detailed analysis showed that Nectin-4 was exclusively expressed in carcinoma cells and absent from myoepithelial and stromal cells (data not shown). IHC showed a cytoplasmic and discrete membrane localization of Nectin-4 (Fig. 2A and 2B). Immunofluorescence studies confirmed IHC and revealed a cytoplasmic expression of Nectin-4 and a clear localization at intercellular junctions between carcinoma cells (Fig. 2C, white arrow). These data show that Nectin-4 is a new marker for breast carcinoma and that its expression strongly correlates with the ductal histological type. Moreover, Nectin-4 expression was not detected in normal breast epithelium suggesting that it can represent a new tumor-associated antigen.

### Nectin-4 expression correlates with basal breast markers

A molecular taxonomy of breast cancers has been proposed that may be related to the differentiation model of breast epithelium. This taxonomy has been well established by gene expression profiling studies [3,4,6]. Studies attempting to define breast cancer subtypes by protein expression profiling have also been reported [22,26,27]. Although very informative, the latter approach needs the characterization of new reliable immunohistochemical markers.

Expression of Nectin-4 was compared with the expression of protein markers on 52 early stage breast tumors. Proteins were selected to identify either basal (EGFR, P53 and P-cadherin) or luminal subtype (ER, PR, GATA3) [26,29,30]. In addition, expression of ERBB2 and of the proliferation marker Ki67 were also analyzed. Expression of E-cadherin, β-catenin and AF6/Afadin, functionally associated with Nectin physiology, were also tested.

**Figure 3:**
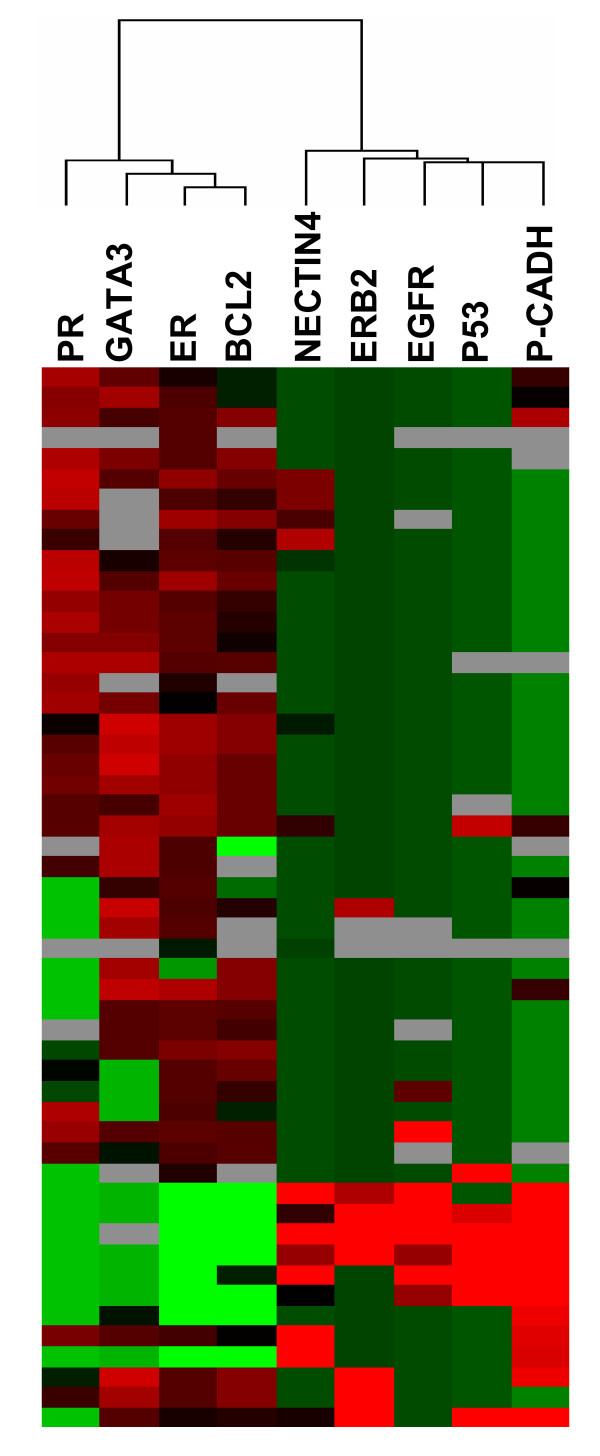
**Nectin-4 expression correlates with basal-like breast markers**. Graphical representation of hierarchical clustering results based on expression profiles of 9 proteins in 52 early breast cancer samples measured by immunohistochemistry. Rows: proteins; columns: samples. Protein expression scores are depicted according to a color scale: red, positive staining; green, negative staining; grey, missing data. Dendrograms represent overall similarities in protein expression profiles. Two clusters of proteins are described. A group of proteins, including Nectin-4, which present high expression profile similarities with basal breast markers. A group of proteins, including luminal markers, which negatively correlate with Nectin-4 expression and basal-like markers.

The overall expression patterns for the 52 samples were analyzed with hierarchical clustering and displayed in a color-coded matrix (Fig. 3). Clustering ordered proteins on the horizontal axis, and tumor samples on the vertical axis on the basis of similarity of their expression profiles. This similarity is shown as a dendrogram in which the length of the branches between two elements reflects their degree of relatedness. A color scale indicates the intensity of protein expression from red for strong staining to green for negative staining. Nectin-4 was identified in a cluster of proteins with EGFR, P53 and P-cadherin (Fig. 3, orange cluster and Table 1). As seen in Table 1, there was strong correlation between Nectin-4 and expression of these markers. Nectin-4 expression was negatively correlated with expression of proteins that define the luminal cluster (ER, PR, GATA3) (Fig. 3, blue cluster and Table 1). A significant, although lower correlation, was noted with ERBB2, Ki67, E-cadherin and β-catenin. More generally, Nectin-4 expression was found in 7 out of 8 of ER/PR-negative tumors. No correlation was noted with AF6/Afadin. Altogether, our data described Nectin-4 as a new protein marker for ductal breast carcinoma. Nectin-4 expression correlates with the expression of proteins that define the basal-like subtype.

### Nectin-4 is a new serological marker for metastatic breast carcinoma

We recently showed that transmembrane Nectin-4 is shed from cell surface, leading to the release of the ectodomain in the culture supernatant. This soluble form of 43.5 kDa comprises the three Ig domains of Nectin-4 (V, C, C). The endoprotease TACE/ADAM-17 cleaves Nectin-4 close to the plasma membrane [28]. Circulating forms of cell adhesion molecules have been reported in different diseases, especially in cancer. However, only two serum markers (CEA and CA15.3) are commonly used to search for MBC but they fail to detect metastasis in all patients and false positivity has been reported.

We thus looked for the presence of a Nectin-4 circulating form in the sera of patients with breast carcinoma. This analysis was performed by ELISA using the N4.40 and the N4.61 mAbs.

**Figure 4:**
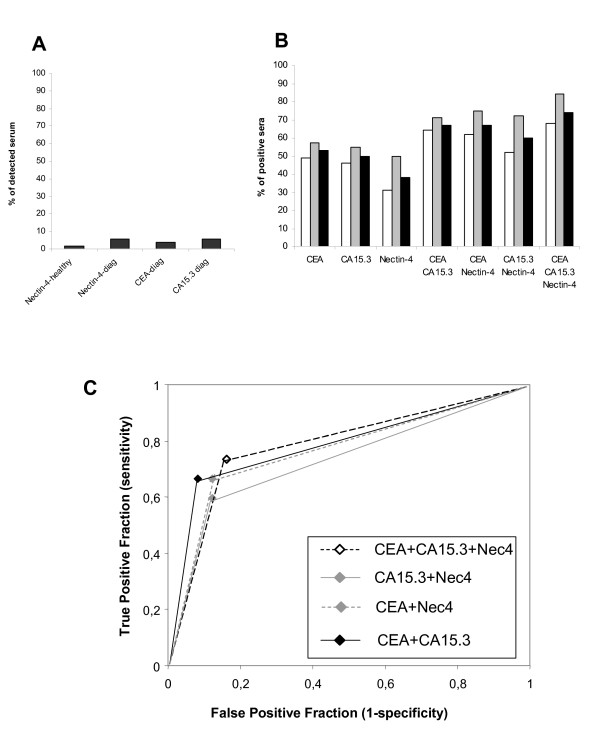
**Detection of Nectin-4 in serum from patients with MBC**. Serum Nectin-4 was detected by ELISA using a pair of anti-Nectin-4 mAbs. **A: **Percentage of Nectin-4 positive sera in healthy donors (n = 45), and in patients with non-metastatic breast tumors compared with CEA and CA15.3 levels (n = 53). **B: **Percentage of sera positive for Nectin-4, CEA and CA15.3 and combinations of markers. The association of Nectin-4 with CEA and CA15.3 increases the percentage of patients detected. White bars; patients with metachronous MBC. Grey bars; patients with synchronous MBC. Black bars; percentage including both groups. **C: **ROC curves were calculated to estimate the accuracy of the association of these markers in breast cancer diagnosis.

First, we confirmed the presence of soluble Nectin-4 in the culture supernatants of Nectin-4 positive cell lines (e.g. T47D, MCF-7, MDA-MB-175) and the absence of this soluble form in the culture supernatants of Nectin-4 negative cell lines (MDA-MB-231 and MDA-MB-134) (data not shown). Second, we found that the soluble form of Nectin-4 was undetectable in 44 of 45 normal sera (Fig. 4A). Third, we searched for soluble Nectin-4 in 53 sera of patients with non-MBC at diagnosis. Two and three patients were positive for CEA and CA15.3 markers, respectively. Five sera presented Nectin-4 levels above the threshold and among them, one serum was also found positive for CEA and CA15.3 markers (Fig. 4A).

We extended the analysis to a panel of 182 sera of patients with MBC and compared the results with the levels of CEA and CA15.3 markers. As shown in Fig. 4B (black bars), circulating forms of Nectin-4 were detected at various levels (up to 1000 pM (125 ng/ml), data not shown), in 38% of tested sera (n = 182). In these same samples, CEA and CA15.3 markers were detected in 53% and 50% of sera, respectively. Interestingly, whereas the combination of the two latter markers reaches 67% detection, the association of CEA, CA15.3 and Nectin-4 allowed 74% detection (Fig. 4B). The increase in sensitivity, assessed by ROC curves, is concomitant with a moderate loss of specificity (from 0.92 to 0.84) (Fig. 4C). Sensitivity and specificity for CEA+Nectin-4 and CA15.3+Nectin-4 were similar to CEA+CA15.3.

Analysis was carried out in the two groups of patients with MBC: patients with synchronous MBC (Fig. 4B, grey bars) or with metachronous MBC (Fig. 4B, white bars). A higher percentage of positive patients was detected for serum Nectin-4 (and also for CEA and CA15.3) in the synchronous MBC group. These data probably highlight the different clinical evolution of these two groups, i.e., a rapid evolution and adverse prognosis of synchronous MBC compared to metachronous MBC.

Our data thus show that Nectin-4 is new reliable serum marker for patients with MBC and can improve patient follow-up.

### Serum Nectin-4 is a marker of disease progression and therapeutic efficiency

**Figure 5:**
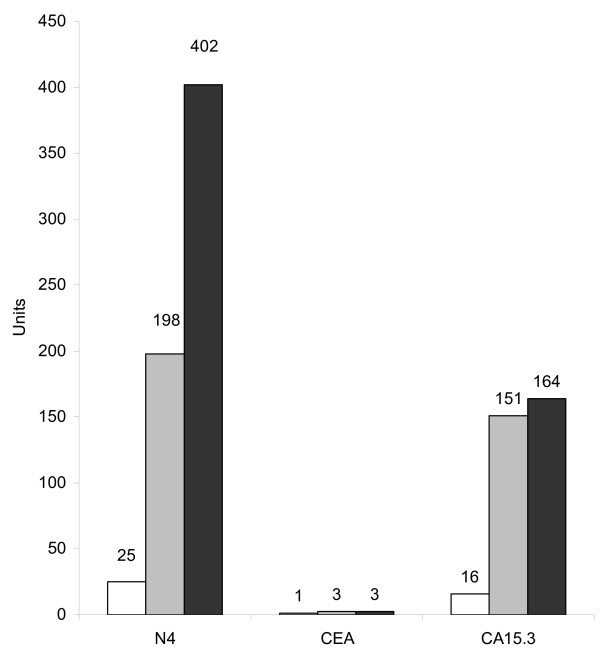
**Serum Nectin-4 and disease progression**. Nectin-4, CEA and CA15.3 serum levels were determined during disease progression of a patient with a ductal carcinoma. The three markers were not detected at the time of diagnosis (white bar). During the progression of the disease, Nectin-4 serum levels increased to reach 402 pM. Grey, detection of pulmonary metastasis 32 months post-diagnosis; black, detection of brain metastasis 39 months post-diagnosis. Ordinates represent pM for Nectin-4 and International Units for CEA and CA15.3.

CEA and CA15.3 markers are classically used to follow the evolution of patients after first remission [31]. The main interest is the possibility to anticipate the clinically observable recurrence. We tested whether detection of soluble Nectin-4 may be used to monitor breast cancer progression. We therefore selected patients presenting increased levels of CEA or CA15.3 during disease progression. As examplified in Fig. 5, high serum levels of Nectin-4 (198 pM) and CA15.3 (151 U/ml) were detected in a patient concomitantly with the appearance of pulmonary metastasis 32 months after diagnosis (gray bars). This patient developed cerebral metastases 39 months after diagnosis (black bars). Whereas CA15.3 levels slightly increased (151 to 164 U/ml), circulating Nectin-4 levels increased two-fold (198 to 402 pM) (16 fold from level at diagnosis). CEA levels were under the threshold value for this patient. These results indicate that Nectin-4 represents a valuable marker for evaluate disease progression. To strengthen this observation, we analyzed the correlation between Nectin-4 levels and the metastatic status of patients based on the clinical detection at diagnosis of either a unique or multiple metastatic localizations. We found that the detection of serum Nectin-4 at diagnosis, correlates with the presence of multiple metastasis in the group of patients with synchronous MBC (*P *= 0.038) (Table 2), and metachronous MBC (*P *= 0.014) (data not shown). Even though CEA and CA15.3 can serve as disease progression markers, we found no significant correlation with the metastasis clinical status (Table 2 and data not shown). No correlation was found between serum Nectin-4 and tumor size, SBR grade, axillary lymph node or ER/PR status (data not shown). These data strongly associate the presence of Nectin-4 in sera to disease progression and to the metastatic status of patients.

**Figure 6:**
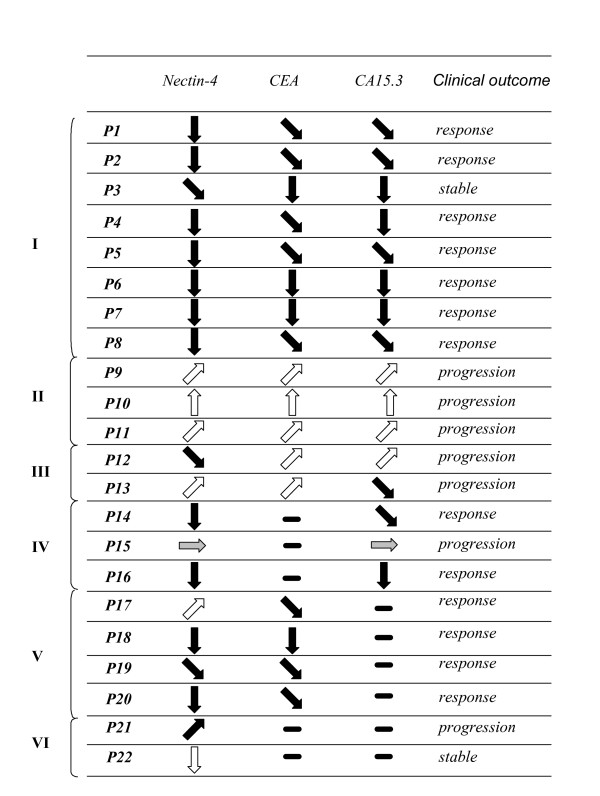
**Serum Nectin-4 and therapeutic efficiency**. Nectin-4, CEA and CA15.3 levels were determined in the serum of patients at the time of metastasis diagnosis and after therapy. Results are represented by arrows that symbolize increased, apparition, decreased, extinction, or unchanged () levels of Nectin-4, CEA and CA15.3. Patient outcome was evaluated according to clinical and histoclinical criteria.

Markers can also be used to measure therapeutic efficiency after treatment of the metastatic disease. We retrospectively analyzed the presence of Nectin-4, CEA and CA15.3 in the sera of 64 patients with metachronous MBC at the time of the metastatic diagnosis and after treatment. Delays between two blood samplings varied from one to nine months. Six groups were defined according to the combination of markers found positive and the clinical outcome (Fig. 6). Groups I and II (patients 1 to 11) correspond to patients with detectable amount of the three markers in their serum. Patients 1 to 8 showed a decrease concomitant with a clinical response in seven cases and a stable disease in one case; patients 9 to 11 showed an increase concomitant with disease progression. Group III included two patients in progression (P12 and P13) where Nectin-4 and CA15.3 levels slightly decreased (see supplementary data). Groups IV, V and VI (patients P14 to P22) corresponded to groups in which Nectin-4 was detected in patients positive for either CEA or CA15.3 or negative for both markers. Nectin-4 levels followed clinical outcome in 90% of cases (20/22).

## Discussion

### Nectin-4 as a potential basal marker in ductal breast carcinomas

The emerging molecular classification of breast cancer is based on data obtained from gene and protein expression profilings. Transcriptome analysis using DNA arrays has divided breast tumors into five subtypes [3,4]. This subtyping contributes to the understanding of the molecular complexity of breast tumors but is difficult to adapt to routine use. Proteome analyzes, e.g. using IHC, has also helped in defining molecular and prognostic signatures of breast cancer and may be easier to use in a clinical setting [22]. Thus, defining new protein markers that help detecting and classifying breast cancer is a priority.

We identified Nectin-4 as a new breast tumor marker. Nectin-4, non expressed in normal cells, is prominently expressed in breast carcinoma cell lines and in breast tumor samples. We found that Nectin-4 expression is associated with markers that define the basal-like subtype of breast cancer. Recently, a molecular classification of breast tumor cell lines has been reported. Among this classification, we found that Nectin-4 is mainly expressed in tumor cell lines with a luminal-like phenotype (BT-483, ZR-75-30, SUM-185, SUM-52, SK-BR-3, MDA-MB-453, HCC1500, ZR-75-1, BT-474, UACC-812, T47D, MCF-7) and absent or weakly expressed in tumor cell lines with a basal-like phenotype (HCC1937, SUM-149, MDA-MB-175, 184B5, MCF-10A, HCC38, BT20, SK-BR-7, MDA-MB-231, MDA-MB-157, Hs578T, BRCa-MZ-01) [26]. These results have been obtained using two different mAbs and validated at the mRNA level. To further document this intriguing point, we looked for other genes with inverse correlation between tumors and cell lines for basal/luminal subtypes, in our gene expression profiling data. Among 47.000 transcripts and variants present on the array, we found 11 genes presenting an inverse signature: the breast stem cell marker CD24, the junctional adhesion molecule JAM-A and the EPHB3 receptor. Interestingly, we found three reports that define CD24 as a luminal-like protein in breast tumor cell lines [32], as a marker whose expression is close to the basal-like gene cluster [33] or whose expression falls into the ER/PR-negative cluster [34], in breast tumors. Thus, CD24 expression does not correlate with the luminal subtype in tumors but rather with markers associated with the basal subtype. Even though these data unambiguously confirm ours, the significance of this set of genes is not clear and will need further investigation. They may be genes particularly involved in stroma-epithelium interactions.

Nectins belong to a family of cell adhesion molecules that regulate the formation and maintenance of adherens junctions in epithelial cells through the AF-6/afadin scaffold molecule [11,12,35]. The Nectin/Afadin complex indirectly interacts with the E-cadherin/β-catenin complex. Loss of Afadin is associated with poor outcome for patients with breast carcinoma [19]. Nectin-4 interacts with Afadin but no significant correlation has been found between the expression of these two proteins in breast carcinoma (*P *= 0.706) [12]. By contrast, we found a correlation between E-cadherin (*P *= 0.0113) and β-catenin (*P *= 0.0231). These results are in accordance with the absence of E-cadherin in most lobular carcinomas [36]. However, we found E-cadherin expression in 95% of ductal carcinomas vs 61% for Nectin-4. E-cadherin is expressed in all subtypes of ductal cancers, whereas Nectin-4 is only expressed in a subset of ductal carcinomas that express basal-like markers.

A major interest of Nectin-4 in breast cancer physiopathology is that this protein is undetectable in normal breast epithelium by IHC. Thus, Nectin-4 is a new tumor-associated antigen and is a candidate for passive and active immunotherapy. However we reproducibly detected a low level of Nectin-4 mRNA in normal breast epithelium (data not shown). This difference could theoretically be explained by i) a lack of sensibility of mAbs, ii) a post-transcriptional regulation of Nectin-4 in normal epithelia, iii) an active cleavage of transmembrane Nectin-4 by TACE protease, iv) the expression of Nectin-4 in a discrete population of cells within the mammary gland. However, we found i) that our mAbs display a good sensitivity in ELISA experiments (Fig. 4), ii) a good correlation between Nectin-4 mRNA and protein level in breast cancer cell lines (Fig. 1C), and iii) that the TACE protease is weakly expressed in the normal mammary gland [28]. Since Nectin-4 expression clusters with basal markers, it is conceivable that Nectin-4 could be present in a rare subset of progenitor cells from which these tumors develop.

### Nectin-4 as a serological marker for breast carcinomas

We and others have found a strong upregulation of TACE protease active form in breast tumors [28,37]. We recently deciphered the mechanism by which Nectin-4 is processed by TACE and showed that soluble Nectin-4 is released as a 43.5 kDa form in both culture supernatant and serum. Synthetic (TAPI-1) and natural (TIMP-3) TACE inhibitors and RNA interference strongly affect Nectin-4 cleavage [28]. TACE is thus mainly responsible for Nectin-4 cleavage in breast tumors. TACE is involved in numerous shedding processes both in normal and pathological situations. The protease regulates MUC1 cleavage and may contribute to the formation of CA15.3 marker.

Because there is no early marker for breast carcinoma, we looked for the presence of soluble Nectin-4 in the serum of patients with non-MBC. Unfortunately, serum Nectin-4 failed to detect most patients with non-MBC and is thus not a good marker of early breast cancer. Co-expression of Nectin-4 and active TACE in a tumor may not be sufficient to induce the release of detectable levels of Nectin-4 in the serum of patients with non-MBC. A similar observation could be done for CA15.3.

Serum Nectin-4 levels are often associated and vary concomitantly with CEA and CA15.3 levels in patients with MBC. Interestingly, we noted that MUC1 is frequently expressed with Nectin-4 in tumors, which could explain the concomitant presence of both markers in patient sera (data not shown). Frequent concomitant detection of serum Nectin-4 with CEA and/or CA15.3 may suggest that circulating Nectin-4 comes directly from tumor cells and not from an indirect process such as immune response.

Serum Nectin-4 may complement CEA and CA15.3 detection. Nectin-4 can be detected in patients negative for both markers or positive for only one marker and thus represents a complementary marker for breast carcinoma. We showed that serum Nectin-4 is strongly associated with metastatic progression (Fig. 5 and Table 2); this could suggest a key role of this molecule during this process. Finally, we showed that in addition to these properties, serum Nectin-4 is a marker of therapeutic efficiency and could contribute to the improvement of patient follow-up. Prospective analyses will evaluate the sensitivity of this new marker, especially its ability to anticipate the clinically observable recurrence.

We found that 50% of ovarian tumor cell lines express transmembrane Nectin-4. Analysis of the sera of patients with metastatic ovarian carcinoma revealed 1/25 positive serum for Nectin-4 vs 19/25 for CA125 (data not shown). We found 0/23 sera positive for Nectin-4 vs 18/23 for PSA in patients with prostate carcinoma (data not shown). Thus, serum Nectin-4 is not a valuable marker for ovarian and prostate carcinomas. Similar observations were reported for serum mammaglobin, where increased serum levels are mainly detected in metastatic breast carcinomas [38].

Nectin-4 may be a new histological and serological marker in breast carcinoma, but its function is unclear. We previously identified Nectin-1 as the counter-receptor of Nectin-4 [25]. Nectin-1 activation leads to the activation of RAC and CDC42 which are involved in the formation of lamellipodia and filopodia respectively. Nectin-4 could promote migratory processes in breast cancer physiopathology.

## Conclusion

In this study, we investigated the potential role of Nectin-4 as a new histological and serological tumor associated marker for breast carcinomas. Nectin-4 and serum Nectin-4 should serve as a new taxonomic, prognosis, and follow-up marker for breast cancer.

## Abbreviations

FACS = Fluorescence activated cell sorter, PCR = Polymerase chain reaction, MBC = metastatic breast carcinoma.

## Competing interests

The author(s) declare that they have no competing interests.

## Authors' contributions

This study was conceived by SFL and ML. Tumor and serum specimens were collected at the Institut Paoli-Calmettes under the responsibility of PV. Expression profiling in cell lines was done by SFL, EL and JA. Quantitative PCR was carried out by CP. Immunohistochemical experiments were performed by CG and JG. JJ and ECJ contributed to pathological analysis and interpretation. PF, AG, and FM performed statistical analysis. CEA and CA15.3 data were under the responsibility of RS, Nectin-4 ELISA were performed by SGU, CB and ML. The manuscript was written by ML with the help of DB, AG, SFL, SGU, ECJ, NM and PD. All authors read and approved the final manuscript.

## Pre-publication history

The pre-publication history for this paper can be accessed here:



## Figures and Tables

**Table 1:** Correlation between histoclinical markers and Nectin-4 expression determined by IHC

***Characteristics***		***N° of patients (%)***		***P-value***
			
		*Nectin4 negative ***(N=39**) 68.4%	*Nectin4 positive ***(N=18) **31.6%	
*Age*	<50	5 (14)	4 (29)	NS
	>50	31 (86)	10 (71)	
				
*Histological type*	Ductal	10 (26)	16 (89)	<0.0001
	Lobular	29 (74)	2 (11)	
				
*SBR grade*	I	9 (25)	3 (21)	NS
	II	17 (47)	6 (43)	
	III	10 (28)	5 (36)	
				
*Axillary lymph node st.*	Negative	19 (53)	6 (43)	NS
	Positive	17 (47)	8 (57)	
				
*EGFR*	Negative	31 (94)	7 (54)	0.0012
	Positive	2 (6)	6 (46)	
				
*ERBB2 status*	0-1	34 (94)	10 (71)	0.0245
	2-3	2 (6)	4 (29)	
				
*Ki67*	Negative	23 (79)	7 (50)	0.05
	Positive	6 (21)	7 (50)	
				
*P53 status*	Negative	33 (97)	7 (50)	<0.0001
	Positive	1 (3)	7 (50)	
				
*P-cadherin*	Negative	25 (74)	4 (29)	0.0029
	Positive	9 (26)	11 (71)	
				
*BCL2*	Negative	2 (6)	6 (43)	0.0025*
	Positive	30 (94)	8 (57)	
				
*Estrogen receptor st.*	Negative	0 (0)	7 (50)	<0.0001*
	Positive	37 (100)	7 (50)	
				
*GATA3*	Negative	3 (9)	6 (60)	0.0004*
	Positive	31 (91)	4 (40)	
				
*Progesterone receptor st.*	Negative	8 (24)	8 (57)	0.0247*
	Positive	26 (76)	6 (43)	

* Negative correlation with Nectin-4 expression

**Table 2:** Correlation between serum Nectin-4, CA15.3, CEA with metastatic status.

***Characteristics***	***N° of patients (%)***		***P-value***
		
	*Nectin4 ≤ 30 ***(N = 51**) 76.1%	*Nectin4 > 30 ***(N = 16) **23.9%	
*Multiple metastasis*	20 (39)	11 (69)	0.038
*Unique metastasis*	31 (61)	5 (31)	

***Characteristics***	***N° of patients (%)***		***P-value***
		
	*CA15.3 ≤ 50 ***(N = 30**) 44.8%	*CA15.3 > 50 ***(N = 37) **55.2%	

*Multiple metastasis*	10 (33)	21 (57)	0.056
*Unique metastasis*	20 (67)	16 (43)	

***Characteristics***	***N° of patients (%)***		***P-value***
		
	*CEA ≤ 5 ***(N = 30**) 44.8%	*CEA3 > 5 ***(N = 37) **55.2%	

*Multiple metastasis*	12 (40)	19 (51)	0.354
*Unique metastasis*	18 (60)	18 (49)	
